# Removal of a Large Splinter from between the Eye-Lids of a Child Two Years of Age

**Published:** 1882-04

**Authors:** Chas. W. Hickman

**Affiliations:** Augusta, Ga.; Lecturer on Eye, Ear and Throat Diseases in the Medical Department of the University of Georgia, Augusta


### REMOVAL OF A LARGE SPLINTER FROM BETWEEN THE EYE-LIDS OF A CHILD TWO YEARS OF AGE.

BY CHAS. W. HICKMAN, M.D., AUGUSTA, GA.
Lecturer on Eye, Ear and Throat Diseases in the Medical Department of the University of Georgia, Augusta.

The following case having struck me as somewhat remarkable, I thought a report of it might not be altogether uninteresting. The patient, a child two years of age, was brought to me a short time ago by its father, with the statement that ten days previous to his coming, while playing with the other children around a wood-pile, it had accidentally run a large splinter into its eye. For some unaccountable reason, it was thought that the splinter had fallen out, as the child for some days seemed to complain but little. Considerable irritation afterwards setting in, however, the eye becoming swollen, and the child fretful—crying nearly all the time, and totally unable to to sleep, it was concluded to bring her into the city in order that a more thorough examination might be made. When seen, the lids were thick and swollen with a yellowish discharge coming from between them ; in short, a condition in every respect resembling an attack of acute catarrhal conjuctivitis. Upon pulling the lids apart, a portion of the splinter could be seen between the inner canthus and the margin of the cornea, but as more harm than good was likely to result from any attempt to discover its exact situation, as well as the amount of damage already done by it,—inasmuch as the child screamed and resisted every effort at an examination—an anæsthetic was administered, and a large splinter, fully five-eighths of an inch in length and nearly one-eighth of an inch in thickness, was found lying against the globe in a vertical direction, almost prizing the lids apart. Its removal with the forceps, coupled with cold applications and bandaging, soon restored the eye to its natural condition, leaving no evidence whatever that the ball had been in any way injured.

